# Immunological Mechanisms and Therapeutic Strategies in Cerebral Ischemia–Reperfusion Injury: From Inflammatory Response to Neurorepair

**DOI:** 10.3390/ijms26178336

**Published:** 2025-08-28

**Authors:** Zhendong Li, Man Li, Zhi Fang, Haijun Wang

**Affiliations:** 1 Department of Neurosurgery, Union Hospital, Tongji Medical College, Huazhong University of Science and Technology, Wuhan 430022, China; 2 Department of Neurology, Union Hospital, Tongji Medical College, Huazhong University of Science and Technology, Wuhan 430022, China

**Keywords:** cerebral ischemia–reperfusion injury, neuroinflammation, immune cell, blood–brain barrier, inflammasome

## Abstract

Cerebral ischemia–reperfusion injury (CIRI) is a complex pathological process that arises when blood flow is restored to the brain after ischemia, often resulting in significant neuronal damage and triggering secondary inflammatory responses. This review explores the immune mechanisms underlying CIRI, focusing on the activation and polarization of resident central nervous system (CNS) cells—particularly microglia and astrocytes—and the infiltration of peripheral immune cells such as neutrophils, monocytes/macrophages, and T lymphocytes. We discuss the central role of microglia in the neuroinflammatory cascade, their polarization between pro-inflammatory (M1) and anti-inflammatory (M2) phenotypes, and how this process influences neuronal damage and tissue repair. This review highlights the roles of the complement system, inflammasome activation, and blood–brain barrier disruption as key drivers of inflammation and neuronal injury. Additionally, we elaborate on the dynamic interactions between resident and infiltrating immune cells, which amplify inflammation and impede post-ischemic recovery. Finally, we discuss emerging therapeutic strategies targeting immune modulation, including cytokine regulation, microglial reprogramming, and targeted drug delivery systems, which offer promising avenues for improving outcomes in ischemic stroke.

## 1. Introduction

Cerebral ischemia—reperfusion injury (CIRI) is a complex pathophysiological event characterized by acute brain tissue damage following the restoration of blood flow after a period of interruption or reduction [1]. This condition often results in irreversible neuronal death and initiates a cascade of secondary harmful events. Cerebral ischemia may result from various factors, including vascular occlusion (e.g., atherosclerosis and thromboembolism), hypertension-related cerebrovascular changes, and acute conditions such as cerebral arterial spasm or hypotension, all of which can disrupt cerebral blood flow [2]. Restoration of blood flow following these ischemic events marks the reperfusion phase.

In clinical practice, cerebral reperfusion is primarily achieved through two established approaches: intravenous thrombolysis and mechanical thrombectomy. Intravenous thrombolysis with recombinant tissue plasminogen activator has long been considered the standard of care, whereas Tenecteplase—a genetically modified variant with an extended half-life and simplified administration—has recently emerged as a promising alternative [2]. Mechanical thrombectomy, which entails endovascular retrieval of occlusive thrombi, has demonstrated superior efficacy in patients with large-vessel occlusion [3]. However, most preclinical models (e.g., middle cerebral artery occlusion) fail to recapitulate pharmacological thrombolysis, as reperfusion is generally achieved through mechanical withdrawal of the obstruction rather than enzymatic clot dissolution [4]. This discrepancy highlights a critical limitation in the translational relevance of current experimental models.

Although reperfusion therapy is the cornerstone of cerebral ischemia treatment, the long-term prognosis for patients with CIRI remains poor [5]. CIRI includes the initial ischemic insult, which is primarily characterized by cell death [6], followed by a series of pathological cascades induced by reperfusion. These cascades involve oxidative stress [7], inflammatory responses [8], mitochondrial dysfunction [7,9], excitotoxicity [10], and calcium overload [11], all contributing to further tissue damage. Accumulating evidence indicates that inflammation plays a central role in CIRI [12]. Ischemic events activate innate immune responses, especially in glial cells, resulting in the release of pro-inflammatory cytokines and reactive oxygen species (ROS) [13,14]. These mediators worsen blood–brain barrier (BBB) disruption, promote neuronal apoptosis, and intensify neuroinflammation [15]. Pro-inflammatory mediators also recruit peripheral immune cells [16], including neutrophils, monocytes/macrophages, and lymphocytes, which infiltrate brain tissue through the disrupted BBB [17,18]. These infiltrating cells recognize exposed central nervous system (CNS) antigens, activate adaptive immune responses, and contribute to secondary neuronal injury [19]. Notably, previous studies have demonstrated that neuroinflammation in ischemic stroke exerts both deleterious and reparative effects: while it exacerbates neuronal injury, it also contributes to tissue remodeling [20]. This dual nature of neuroinflammation highlights the complexity of post-ischemic immune responses and underscores the need for precise therapeutic modulation to balance injury and repair.

Although extensive research has investigated the complex mechanisms of inflammation in CIRI, a comprehensive perspective on the roles of immune responses and inflammation remains lacking. Understanding these mechanisms is essential as inflammation is central to the pathophysiology of CIRI and may offer insights into novel therapeutic targets and strategies. This review aims to systematically summarize the immune regulatory mechanisms involved in CIRI, with a focus on the interactions among immune cells, signaling pathways, and regulatory strategies. Specifically, we elaborate on immune modulation at different stages of CIRI, the regulation and repair of the BBB, the functions and interactions of various immune and neural cells, and the activation of inflammasomes during inflammation. By integrating existing evidence, this review seeks to provide a comprehensive understanding of immune regulation in CIRI and offers novel perspectives for future research and therapies.

## 2. The Sequential Role of the Immune System in CIRI

### 2.1. Acute Phase

The acute phase of CIRI typically occurs within 24 to 72 h after reperfusion [21]. During this period, the immune system initiates a rapid inflammatory response that critically influences the extent of neuronal injury.

Resident microglia become activated within minutes of ischemia, releasing pro-inflammatory cytokines such as tumor necrosis factor-α (TNF-α) and interleukin-1β (IL-1β) [22], as well as chemokines such as C–C motif chemokine ligand-2 (CCL2) [23]. These mediators exacerbate neuroinflammation and disrupt the BBB, facilitating the infiltration of peripheral immune cells—including neutrophils, monocytes, and lymphocytes—into the brain [21].

Neutrophils, as early responders, exacerbate tissue injury by releasing ROS, proteolytic enzymes, and neutrophil extracellular traps (NETs) [24]. ROS are mainly produced by NADPH oxidase, which triggers oxidative stress, lipid peroxidation, and mitochondrial dysfunction. These processes collectively worsen neuronal death in both the ischemic core and the penumbra [25]. In addition, ROS activate signaling pathways such as nuclear factor-κB (NF-κB), which upregulate adhesion molecules and perpetuate the inflammatory response [26,27].

Upon entering the brain, peripheral monocytes differentiate into macrophages exhibiting either a pro-inflammatory (M1) or anti-inflammatory (M2) phenotype [23]. M1 macrophages promote neuroinflammation by producing pro-inflammatory cytokines, whereas M2 macrophages offer transient neuroprotection through the secretion of anti-inflammatory mediators such as interleukin-10 (IL-10) [28]. However, the reparative function of M2 macrophages is limited by their short lifespan during the acute phase [29].

Similarly, infiltrating T lymphocytes, including CD4+ and CD8+ subtypes, modulate the inflammatory environment. T helper 1 cells (Th1) and T helper 17 cells (Th17) contribute to tissue damage, whereas regulatory T cells (Tregs) suppress inflammation [27]. The complex interactions between resident and infiltrating immune cells create a feedback loop that perpetuates inflammation and worsens secondary injury [6,21].

Although the inflammatory response in CIRI is predominantly harmful, it is essential for clearing damaged tissue. Microglia and macrophages play dual roles depending on their activation state [30]. However, during the acute phase, the predominance of the M1 phenotype often shifts the balance toward neurotoxicity [29]. This complex immune response is regulated by signaling pathways such as NF-κB and mitogen-activated protein kinase (MAPK) [31]. These pathways underscore the dual role of inflammation in CIRI: dysregulated responses exacerbate injury, whereas timely modulation may facilitate recovery.

### 2.2. Subacute Phase

During the subacute phase of CIRI—typically 72 h to 7 days after ischemia [32]—the immune system remains highly active. Dynamic interactions among various immune cells contribute to both inflammatory and reparative processes. However, the overall immune response remains predominantly pro-inflammatory.

Neutrophils remain active in the pro-inflammatory response initiated during the acute phase and peak around day 3, exacerbating oxidative stress, disrupting the BBB, and contributing to secondary injury [33]. However, neutrophils also contribute to repair by releasing proteases that remodel the extracellular matrix, thereby promoting angiogenesis and tissue regeneration [34].

Microglia, the resident immune cells of the CNS, are rapidly activated during this phase and predominantly assume a pro-inflammatory M1 phenotype. M1 microglia release pro-inflammatory cytokines—including TNF-α, IL-1β, and interleukin-6 (IL-6)—which exacerbate neuronal injury, oxidative stress, and inflammation, thereby enlarging the ischemic core [35,36]. However, mechanisms such as interleukin-4 (IL-4) signaling promote the polarization of microglia toward the anti-inflammatory M2 phenotype, which facilitates debris clearance, suppresses inflammation, and promotes tissue repair [37].

Astrocytes also play a key role in the subacute phase by undergoing reactive astrogliosis, characterized by structural and functional alterations. While reactive astrocytes help to preserve BBB integrity and secrete neurotrophic factors, they may also shift toward the neurotoxic A1 phenotype, releasing neurotoxic molecules (e.g., complement component C3) that impair synaptic repair and neuronal survival [38,39]. Additionally, astrocyte activation may lead to glial scar formation, which physically impedes axonal regeneration and delays functional recovery [40,41]. Interactions between microglia and astrocytes establish a feedback loop that amplifies neuroinflammation [42].

Lymphocytes (primarily T cells) are recruited to ischemic brain tissue during the subacute phase, where they exert both detrimental and protective effects. CD4^+^ T cells—particularly Th1 cells—secrete pro-inflammatory cytokines such as interferon-γ (IFN-γ) and TNF-α, thereby aggravating neuroinflammation and tissue injury [43]. However, Tregs begin to accumulate during this phase and secrete anti-inflammatory cytokines such as IL-10 and transforming growth factor-β (TGF-β), which modulate the immune response and facilitate early tissue repair [44]. In contrast, CD8^+^ cytotoxic T cells may induce neuronal death by targeting neurons presenting antigens through major histocompatibility complex (MHC) class I molecules [45].

The subacute phase is marked by intense inflammation that not only causes tissue damage, but also initiates repair processes. During this phase, maintaining a balance between pro- and anti-inflammatory responses is essential to limit the progression of injury and support subsequent recovery.

### 2.3. Chronic Phase

In the chronic phase, which typically extends beyond six weeks [46], the immune response shifts toward inflammation resolution and tissue repair.

During this phase, microglia polarize toward the M2 phenotype and secrete anti-inflammatory cytokines (including IL-10 and TGF-β) which facilitate neurogenesis, angiogenesis, and tissue repair [47].

Similarly, astrocytes polarize toward the anti-inflammatory A2 phenotype and release growth factors—including brain-derived neurotrophic factor (BDNF) and vascular endothelial growth factor (VEGF)—to promote neuronal survival and vascular remodeling [39].

Notably, oligodendrocytes play a vital role in the chronic phase by promoting myelin regeneration and restoring white matter integrity, which is essential for cognitive recovery [39]. However, persistent low-grade inflammation—commonly termed “inflammaging”—may hinder these reparative processes, highlighting the importance of immune regulation [48,49].

Lymphocytes remain actively involved in the chronic phase. Tregs accumulate in the ischemic brain, where they suppress excessive inflammation and modulate astrocyte activity, thereby attenuating astrogliosis and glial scar formation, ultimately promoting neural repair [44]. Th2 cells secrete IL-4 and IL-10, further facilitating inflammation resolution and enhancing tissue repair [50].

The chronic phase is characterized by repair and resolution, during which the immune response gradually promotes tissue regeneration and functional recovery. However, persistent low-grade inflammation may disrupt these processes, underscoring the need for strategies that enhance repair while effectively suppressing chronic inflammation.

## 3. The Interaction Between the BBB and Immune Regulatory Mechanisms

**Figure 1:**
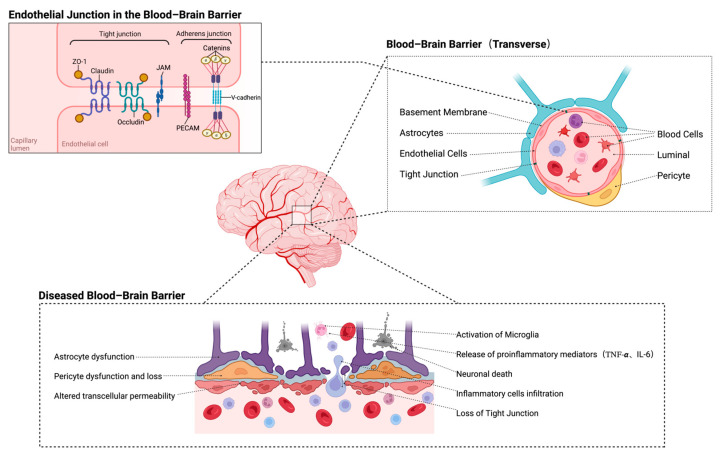
**The composition and structural organization of the BBB.** The BBB is a selective barrier composed of endothelial cells connected by tight and adherens junctions and supported by pericytes and astrocytic endfeet. BBB dysfunction can lead to increased permeability and subsequent neuroinflammation (the figure is generated via BioRender).

The BBB, a highly specialized and dynamic interface (Figure 1), is a fundamental component of the neurovascular unit (NVU) which plays a pivotal role in maintaining CNS homeostasis [51]. Structurally, the BBB consists of brain microvascular endothelial cells interconnected by tight junctions and adherens junctions, and is supported by pericytes, astrocytic endfeet, and the extracellular matrix [52,53]. Tight junction proteins—including claudin-5, occludin, and zonula occludens-1 (ZO-1)—regulate paracellular permeability, while adherens junctions such as vascular endothelial-cadherin (VE-cadherin) contribute to structural stability and mechanical support [54]. Pericytes and astrocytes are key regulators of BBB function. Astrocytes release neurotrophic factors, including VEGF and TGF-β, to maintain endothelial integrity, while pericytes modulate endothelial behavior and interactions with the extracellular matrix [55,56]. The extracellular matrix offers both structural support and biochemical cues which are essential for maintaining BBB stability [53]. This intricate architecture underpins the BBB’s role as a gatekeeper, protecting the CNS by tightly regulating molecular transport, limiting immune cell infiltration, and preserving an optimal microenvironment for neuronal function.

During CIRI, the BBB is rapidly disrupted, initiating a cascade of pathological events. Within minutes of ischemia and subsequent reperfusion, oxidative stress, inflammation, and metabolic dysfunction lead to the degradation of tight junction proteins, disruption of the actin cytoskeleton, and increased transcytosis via caveolin-1-dependent vesicular transport [57,58]. These events increase the BBB’s permeability, allowing plasma proteins, water, and immune cells to extravasate into the brain parenchyma [59]. The resulting vasogenic edema, neuroinflammation, and neuronal injury define the acute phase of BBB disruption [52]. Early recruitment of immune cells, including neutrophils and monocytes, is driven by the upregulation of adhesion molecules such as intercellular adhesion molecule-1 (ICAM-1) and vascular cell adhesion molecule-1 (VCAM-1) on endothelial cells [60,61]. These infiltrating immune cells release ROS, pro-inflammatory cytokines (e.g., TNF-α, IL-6), and matrix metalloproteinases (MMPs), further exacerbating endothelial injury and worsening BBB dysfunction [17,58,62]. As injury progresses, the inflammatory response peaks within 24–48 h after reperfusion and transitions into a secondary phase characterized by sustained MMP activity, extracellular matrix degradation, and ongoing immune cell infiltration [56]. Prolonged inflammation further aggravates BBB disruption and contributes to chronic neuroinflammation [52]. Key inflammatory signaling pathways, including NF-κB and signal transducer and activator of transcription (STAT) 3, contribute significantly by enhancing cytokine production, promoting endothelial apoptosis, and increasing leukocyte adhesion and transmigration [60,63]. Collectively, these mechanisms establish a self-perpetuating cycle of injury, leading to sustained BBB instability and worsening neuronal damage and functional deficits.

Despite extensive damage during CIRI, the BBB demonstrates a remarkable capacity for repair through coordinated interactions among NVU components. Astrocytes play a central role in this process by secreting factors such as VEGF, TGF-β, and thrombospondins, which promote angiogenesis, endothelial survival, and tight junction reassembly [53,55]. Pericytes stabilize nascent blood vessels, regulate endothelial barrier function, and interact with the extracellular matrix to restore vascular integrity [54]. Microglial polarization from the M1 to M2 phenotype supports recovery by attenuating chronic inflammation, promoting tissue remodeling, and establishing a permissive environment for neurovascular repair [17,64]. Endothelial cells actively contribute to BBB repair by re-expressing tight junction proteins and reorganizing their cytoskeleton to restore barrier integrity [65]. The extracellular matrix provides structural support and biochemical cues for cell adhesion and migration during BBB repair [66]. As inflammation resolves, astrocytes and pericytes help to re-establish selective permeability in the BBB, thereby restoring a homeostatic microenvironment that supports neuronal survival and recovery [67]. These repair mechanisms highlight the BBB’s dynamic and adaptive nature, underscoring its pivotal role in post-ischemic recovery.

The BBB is crucial for CNS homeostasis but becomes disrupted during CIRI, permitting immune infiltration and aggravating neuronal injury. Despite this, astrocytes, pericytes, and endothelial cells coordinate BBB repair and support recovery. Understanding these mechanisms may guide therapies to preserve or restore BBB integrity in the context of ischemic stroke.

## 4. Resident Immune Cells and Astrocytes in CIRI

Although the brain is considered immune-privileged, resident immune cells (microglia) and astrocytes in CNS are essential for maintaining homeostasis and neuroprotection. In CIRI, they rapidly respond to injury and become highly activated, highlighting their key regulatory roles. The following sections explore their functions and activation mechanisms in the pathophysiology of CIRI.

### 4.1. Microglia

#### 4.1.1. Physiological Role of Microglia in the CNS

Microglia, the resident immune-competent cells of the CNS, play a vital role in maintaining brain homeostasis and coordinating innate immune defense mechanisms [68]. Originating from yolk sac macrophages during early embryogenesis, microglia migrate into the neuroepithelium prior to BBB formation, distinguishing them from circulating monocytes that infiltrate the CNS under pathological conditions [68,69]. In a healthy CNS, microglia are evenly distributed across brain regions and remain highly dynamic, constantly extending and retracting their processes to surveil the microenvironment for disturbances.

Microglia perform essential immune and non-immune functions to maintain stability of the CNS. A key function of microglia is phagocytosis, which enables the clearance of apoptotic cells, cellular debris, and invading pathogens, thereby preserving tissue integrity and preventing inflammation [70]. Microglia express a diverse repertoire of receptors, including toll-like receptors (TLRs), complement receptors, and triggering receptors expressed on myeloid cells-2 [71], allowing them to detect and respond to molecular signals of stress or injury. Through these receptors, microglia promote the clearance of cellular waste, mediate communication with other glial cells, and coordinate local immune responses to maintain CNS homeostasis.

In addition to their immune functions, microglia play a significant role in supporting neural cells. They secrete neurotrophic factors—including insulin-like growth factor-1 and BDNF [72]—which promote neuronal survival, support myelin integrity, and facilitate tissue repair. Additionally, microglia interact with astrocytes and endothelial cells to preserve the structural and functional integrity of the BBB, a critical interface that protects the CNS from peripheral immune infiltration and toxic substances [73].

Notably, microglia exhibit region-specific functional adaptations within the CNS, allowing them to meet the distinct demands of different brain regions. For example, their ability to regulate inflammation and mediate neuroprotection varies according to the local molecular and cellular environment [70]. As demonstrated in the studies of Colonna et al. and Butovsky et al., microglia exhibit limited antigen-presenting capacity under physiological conditions, characterized by low expression of MHC class II and costimulatory molecules [69]. This feature reflects their role in maintaining immune surveillance while avoiding excessive immune activation, a hallmark of CNS immune privilege.

#### 4.1.2. Microglia in CIRI

Microglia play a pivotal role in the inflammatory response to CIRI, acting as the primary immune responders within the CNS. Following ischemic insult and reperfusion, microglia rapidly activate, shifting from a surveillant, ramified morphology to a reactive, amoeboid form [74]. This activation leads to microglial polarization into two distinct phenotypes—pro-inflammatory M1 and anti-inflammatory M2—each exerting opposing effects on injury progression and resolution. M1 microglia release pro-inflammatory mediators such as TNF-α, IL-1β, IL-6, and ROS [75], which exacerbate neuronal apoptosis, oxidative stress, and BBB disruption, thereby amplifying tissue damage [76]. Maida et al. noted that TNF-α and IL-1β are among the earliest cytokines to rise after ischemic stroke, driving leukocyte adhesion, BBB breakdown, and neuronal death, while IL-6 displays context-dependent effects, acting as a pro-inflammatory factor in the acute phase but exerting neurotrophic and reparative functions later [20]. In contrast, M2 microglia secrete anti-inflammatory cytokines such as IL-10 and TGF-β [77] and facilitate the phagocytosis of apoptotic cells and debris, thereby promoting neuroprotection, tissue remodeling, and angiogenesis [74,78]. In addition to cytokine secretion, distinct molecular markers further characterize M1/M2 polarization. M1-polarized microglia typically express CD16/32 and secrete nitric oxide (NO), reinforcing their pro-inflammatory and neurotoxic roles [77]. Conversely, M2-polarized microglia upregulate arginase-1 (Arg1), CD206, suppressor of cytokine signaling-3 (SOCS3), found in inflammatory zone-1 (Fizz1), and chitinase-like protein-3 (Ym1), which are associated with enhanced phagocytosis, tissue repair, and neuroprotection [79,80,81].

Microglial activation and polarization during CIRI are orchestrated by a network of interconnected signaling pathways that balance inflammatory injury and reparative responses. NF-κB serves as a pivotal driver of M1 polarization, which is rapidly activated downstream of TLRs and other pattern-recognition receptors sensing ischemia-induced damage-associated molecular patterns (DAMPs). Canonical NF-κB signaling upregulates pro-inflammatory mediators such as TNF-α, IL-1β, IL-6, and inducible nitric oxide synthase, thereby amplifying oxidative stress, leukocyte recruitment, and neuronal injury. Non-canonical NF-κB activation may sustain inflammatory programs in the subacute phase, while cooperative interactions with stress-activated MAPKs form a feed-forward loop that further enhances cytokine production [82,83,84]. MAPK signaling, particularly via p38 and c-Jun N-terminal kinase, is similarly triggered by DAMPs and upstream receptors, leading to activator-protein-1-dependent transcription of pro-inflammatory cytokines and MMPs such as MMP-9. These events promote endothelial dysfunction, compromise BBB integrity, and facilitate peripheral immune cell infiltration. Collectively, the NF-κB and MAPK pathways are key contributors to the inflammatory axis driving post-ischemic neuroinflammation [76,85]. The Janus kinase (JAK)/STAT axis enables additional regulation of the microglial phenotype. IL-6 family cytokines activate JAK-mediated phosphorylation of STAT3, which dimerizes and translocates to the nucleus. Depending on the disease stage and cellular context, STAT3 may either propagate pro-inflammatory transcription or contribute to neurorepair. Conversely, IL-4-driven STAT6 activation programs microglia toward an M2 phenotype, characterized by Arg1 and CD206 expression, thereby counteracting NF-κB and MAPK-mediated injury responses [85,86]. In contrast, phosphoinositide 3-kinase/protein kinase B (PI3K/Akt) signaling exerts broad cytoprotective and reparative functions. Pathway activation suppresses inflammatory responses, enhances antioxidant defenses, inhibits apoptosis, and promotes M2 polarization with upregulation of Arg1, CD206, IL-10, and TGF-β. Pharmacological activators such as quercetin enhance PI3K/Akt activity, inhibit NF-κB signaling, and augment microglial reparative capacity, whereas pharmacologic blockade abolishes these effects [77,87].

Inflammasomes are key regulators of microglia-mediated neuroinflammation during CIRI. Among them, the pyrin domain-containing 3 (NLRP3) inflammasome is the most extensively studied. Stimulated by ROS, mitochondrial dysfunction, and extracellular ATP, NLRP3 promotes the cleavage of pro-caspase-1 into active caspase-1, which subsequently processes pro-IL-1β and pro-IL-18 into their mature forms. These cytokines amplify the inflammatory cascade by recruiting peripheral immune cells and aggravating neuronal injury [75,77,78]. Additionally, IL-1β perpetuates inflammation by activating downstream signaling via IL-1R1 and TNF receptor-associated factor 6, thereby linking inflammasome activation to the NF-κB and MAPK pathways [88]. Other inflammasomes—including absent in melanoma 2 (AIM2) and NLR family caspase recruitment domain (CARD) domain-containing protein 4 (NLRC4)—also contribute to microglia-driven inflammatory responses during CIRI. AIM2 activation induced by mitochondrial and nuclear damage leads to the release of IL-1β and IL-18, which further compromise BBB integrity and intensify inflammation [89]. Similarly, NLRC4 enhances inflammatory signaling and reinforces microglial responses to ischemic injury [90].

Mitochondrial dysfunction serves as a key upstream regulator of inflammasome activation during CIRI. The mitochondrial DNA (mtDNA) released into the cytosol functions as a potent DAMP, activating inflammasomes such as NLRP3 and AIM2 [89,91]. Concurrently, ROS generated by damaged mitochondria directly activate inflammasomes and amplify downstream inflammatory cascades [92]. Beyond inflammasome activation, cytosolic mtDNA is also sensed by cyclic GMP–AMP synthase (cGAS), which generates cyclic GMP–AMP to activate stimulator of interferon genes (STING). This in turn, recruits TANK-binding kinase 1 and IκB kinase which co-activate interferon regulatory factor 3 and NF-κB, thereby inducing the production of type I interferon and pro-inflammatory cytokines [74,84]. Through these convergent mechanisms, mitochondrial stress links innate immune activation to enhanced M1 polarization during CIRI.

Microglia play a central role in CIRI by dynamically shifting between pro- and anti-inflammatory states. Their activation is governed by pathways such as NF-κB, MAPK, and inflammasomes. Understanding these mechanisms may reveal therapeutic targets to reduce neuroinflammation and promote recovery.

### 4.2. Astrocytes

#### 4.2.1. The Physiological Roles of Astrocytes in the CNS

Astrocytes, the most abundant glial cells in the CNS, perform diverse and essential physiological functions crucial for maintaining CNS homeostasis. A key function of astrocytes is the regulation of neurotransmission, primarily through the uptake and recycling of neurotransmitters such as glutamate and γ-aminobutyric acid. This process is mediated by high expression of transporters such as glutamate transporter-1 and glutamate aspartate transporter on astrocytic processes, preventing excitotoxicity and preserving synaptic function [93]. Additionally, astrocytes contribute to synaptic plasticity and remodeling by releasing neurotrophic factors such as BDNF [94] and thrombospondins [95], which promote synaptogenesis and facilitate repair after CNS injury.

In addition to their role in neurotransmission, astrocytes are essential for maintaining the structure and function of the BBB. They preserve BBB integrity by secreting tight-junction-associated molecules and neurotrophic factors, while their end-feet regulate water and ion homeostasis through channels such as aquaporin-4 and potassium channels [96]. These mechanisms ensure ionic homeostasis and protect against brain edema under both physiological and pathological conditions. Furthermore, astrocytes support neuronal energy metabolism by delivering glucose and lactate to meet the high metabolic demands associated with neuronal activity [95].

#### 4.2.2. Astrocytes in CIRI

Astrocytes are central mediators of inflammation in CIRI, functioning both as initiators of neuroinflammation and contributors to neuronal damage [39]. Following ischemic insult, astrocytes in the affected region undergo reactive astrogliosis, characterized by hypertrophy, proliferation, and the release of inflammatory mediators [97]. Reactive astrocytes polarize into two distinct phenotypes: the neurotoxic A1-like and the neuroprotective A2-like states [98]. A1-like astrocytes, activated by inflammatory signals such as IL-1β, TNF-α, and complement proteins, secrete neurotoxic mediators including ROS, complement component C3, and pro-inflammatory cytokines like IL-6. These mediators contribute to neuronal apoptosis, BBB disruption, and exacerbation of neuroinflammation [99,100,101]. In contrast, A2-like astrocytes exert neuroprotective effects by releasing anti-inflammatory cytokines and neurotrophic factors, including VEGF and TGF-β [102]. These molecules play pivotal roles in stabilizing the BBB, promoting angiogenesis, and supporting neuronal survival, particularly in the later stages of CIRI [102,103]. Moreover, pathogen- or microbe-associated molecular patterns (PAMPs or MAMPs), such as lipopolysaccharide (LPS), as well as IFN-γ stimulation, can drive astrocytic reactivity and synergize with IL-1β to exacerbate neuroinflammatory cascades [104].

Among the mediators of astrocyte-mediated neuroinflammation, TNF-α and IL-1β are key pro-inflammatory cytokines predominantly expressed by A1-like astrocytes [98]. TNF-α signaling via TNF receptor (TNFR) 1 (TNFR1) activates apoptotic pathways, whereas signaling through TNFR2 may promote neuronal survival under specific conditions [100]. Similarly, IL-1β binds to its receptor IL-1R and activates the NF-κB pathway, which promotes sustained production of inflammatory mediators such as cytokines, chemokines, and adhesion molecules [99]. These inflammatory cascades recruit peripheral immune cells to the injury site, thereby exacerbating neuroinflammation and tissue damage. Reactive astrocytes also secrete chemokines such as C-X-C motif chemokine ligand (CXCL)-1 (CXCL1) and CXCL2, which attract neutrophils to the injury site [1,97]. These neutrophils further enhance inflammation by releasing ROS and proteases, thereby exacerbating neuronal damage [105]. Additionally, under NF-κB signaling conditions, astrocytes and endothelial cells upregulate adhesion molecules such as ICAM-1 and VCAM-1, facilitating leukocyte infiltration into the brain parenchyma and further compromising BBB integrity [97,99]. Astrocytes also play a pivotal role in oxidative stress during CIRI. NADPH oxidase activation in reactive astrocytes induces excessive ROS production, leading to cellular damage and exacerbated oxidative stress [106,107]. Moreover, metabolic dysregulation in astrocytes aggravates neuronal injury. Impaired glycogenolysis reduces astrocytic metabolic support to neurons, whereas excessive lactate production induces protein lactylation, thereby promoting inflammation and glial activation [105,108].

In conclusion, astrocytes play dual roles in CIRI, contributing to both injury and repair through reactive phenotypes, signaling cascades, and metabolic functions. Therapeutic strategies aimed at promoting A2-like astrocytic functions while inhibiting A1-like phenotypes, alongside modulation of NF-κB and NADPH oxidase pathways, show promise in attenuating neuroinflammation and enhancing recovery following ischemic stroke.

## 5. Peripheral Immune Cells and Platelets in CIRI

Peripheral immune cells play a crucial role in the pathophysiology of CIRI. Following reperfusion, immune cells, including neutrophils, monocyte-derived macrophages (MoDMs), and T cells, are rapidly recruited to the ischemic brain tissue, where they critically modulate the inflammatory response and contribute to tissue injury. Platelets, traditionally known for their role in hemostasis, have now emerged as key modulators of thrombo-inflammation by interacting with T cells and endothelial cells, thereby amplifying immune responses and contributing to microvascular dysfunction and infarct expansion [109]. As first responders, neutrophils release pro-inflammatory cytokines and proteolytic enzymes, leading to BBB disruption and neuronal damage [6,19]. MoDMs, which originate from peripheral monocytes, participate in apoptotic cell clearance and secrete cytokines such as TNF-α, thereby modulating inflammation and tissue repair [22,110]. T cells—particularly CD4^+^ and CD8^+^ subsets—exacerbate inflammation and neuronal injury, whereas Tregs suppress excessive immune responses and promote tissue repair [27,66,111]. The complex interplay among these immune cells is critical in determining the severity of injury and recovery potential following CIRI.

### 5.1. Neutrophils

Neutrophils, as key effector cells of the innate immune system, play crucial and multifaceted roles in the immunoregulatory mechanisms underlying CIRI. Following ischemic stroke, neutrophils are rapidly recruited to the injured brain, guided by chemokine gradients such as CXCL1, CXCL2, CXCL8, CXCL9, and CXCL10, which bind to the C-X-C chemokine receptor (CXCR) receptor and promote their migration to ischemic regions. This recruitment is further amplified by pro-inflammatory cytokines, including TNF-α and IL-1β [112], which induce the expression of adhesion molecules such as ICAM-1, VCAM-1, and selectins on endothelial cells [113]. These molecular interactions facilitate neutrophil adhesion, transmigration across the BBB, and accumulation within the ischemic tissue [62]. Once in the brain parenchyma, neutrophils contribute to both acute inflammation and secondary brain injury through the release of multiple inflammatory mediators. ROS, MMPs, and pro-inflammatory cytokines further exacerbate BBB disruption, neuronal apoptosis, and neuroinflammation [1,34,114]. Another key mechanism of neutrophil-mediated injury is the formation of NETs, consisting of decondensed chromatin, histones, and granule-derived proteins [115]. Although NETs serve as a host defense mechanism by trapping pathogens, excessive NET formation can aggravate endothelial injury, promote thrombosis, and exacerbate ischemic damage by enhancing local inflammation and impairing vascular reperfusion [116].

Neutrophils exhibit functional plasticity and are capable of polarizing into pro-inflammatory (N1) or anti-inflammatory (N2) phenotypes in response to the local microenvironment. N1 neutrophils aggravate injury through the release of ROS, cytokines, and proteases, whereas N2 neutrophils facilitate tissue repair by promoting angiogenesis, clearing cellular debris, and resolving inflammation [66,117]. This dual role underscores the complexity of neutrophil-mediated responses in CIRI and supports therapeutic approaches that modulate, rather than completely inhibit, neutrophil activity [117]. Beyond their direct effects, neutrophils dynamically interact with other immune cells, thereby shaping the broader inflammatory landscape in CIRI [118]. For example, neutrophils activate microglia and macrophages via cytokines such as TNF-α and IL-1β, establishing a feedforward inflammatory loop that worsens neuronal injury [117]. Conversely, under specific conditions, neutrophils secrete anti-inflammatory mediators such as lipoxin A_4_, contributing to inflammation resolution and tissue repair, thereby reinforcing their dual role [119,120]. Neutrophils also play a central role in the thrombo-inflammatory processes associated with CIRI. Through the formation of platelet–neutrophil aggregates and the release of MMPs, neutrophils exacerbate microvascular obstruction and intensify ischemic injury [121]. Targeting these interactions represents a promising therapeutic strategy to mitigate neutrophil-driven damage while preserving neutrophils’ reparative functions.

Recent advances have identified several molecular pathways that regulate neutrophil activity in CIRI. CD13, a neutrophil-expressed metalloprotease, facilitates transmigration across the BBB and enhances pro-inflammatory activity [122]. In experimental models, inhibition of CD13 reduces neutrophil infiltration, decreases infarct volume, and improves neurological outcomes [122]. Similarly, CXCL12/CXCR4 signaling not only retains neutrophils at the injury site but also promotes tissue repair during recovery, highlighting its potential as a therapeutic target [123].

Neutrophils play a dual role in CIRI, driving acute inflammation and contributing to repair. Their rapid recruitment and interactions with other immune cells highlight their complexity. Targeting NETs, CD13, and key signaling pathways offers promising strategies to reduce neuroinflammation and improve recovery in the context of ischemic stroke.

### 5.2. MoDMs

MoDMs play a critical role in the immune response following CIRI. Similarly to other tissues, the brain contains diverse myeloid-derived populations including resident microglia and injury-induced infiltrating monocytes. Microglia, the brain’s resident macrophages, originate from yolk sac progenitors during early embryogenesis and maintain homeostasis throughout life [124]. In contrast, monocytes originate from the bone marrow and circulate in the peripheral blood [125]. Following ischemic injury, monocytes are rapidly recruited to the brain and differentiate into macrophages [126]. Although microglia and MoDMs share functional similarities in inflammation and repair, they arise from distinct lineages and play fundamentally different roles in the post-stroke brain. Monocytes are classified into three subsets based on surface marker expression: classical, intermediate, and non-classical. Classical monocytes (Ly6Chi in mice, CD14+CD16−- in humans) are primarily recruited to inflamed tissues, where they differentiate into pro-inflammatory macrophages [110]. These macrophages release ROS, cytokines (e.g., TNF-α, IL-1β, and IL-6), and proteases, all of which exacerbate ischemic injury by promoting neuroinflammation and disrupting the BBB [110,127]. In contrast, non-classical monocytes (Ly6Clo in mice and CD14−CD16+ in humans) are associated with the resolution of inflammation and tissue repair [110]. These monocytes differentiate into anti-inflammatory macrophages that secrete cytokines such as IL-4 and IL-10, thereby promoting tissue regeneration through debris clearance and neuronal support [110,125]. The intermediate subset, exhibiting features of both classical and non-classical monocytes, contributes to both inflammation and repair, depending on the context of the injury [110].

The recruitment and differentiation of MoDMs in CIRI are tightly regulated by cytokines and chemokines released from ischemic tissue [126]. A key mediator in this process is CCL2, which binds to CCR2 on monocytes and directs their migration to the ischemic brain [127]. Upon entering the brain, monocytes differentiate into macrophages in response to local cues, including cytokines secreted by microglia and astrocytes [125]. These cues ensure that MoDMs adopt appropriate phenotypes for brain repair, contributing to both inflammation during early injury and tissue regeneration during later recovery phases. In addition to their pro-inflammatory functions, MoDMs contribute to brain repair through efferocytosis—the clearance of apoptotic cells [128]. This process facilitates the removal of dead cells and prevents the release of harmful intracellular contents into surrounding tissue, thereby creating a favorable environment for repair and regeneration [129,130]. By clearing apoptotic neurons and cellular debris, MoDMs support the resolution of neuroinflammation and aid in the restoration of normal brain function after ischemic injury.

The functional plasticity of MoDMs is orchestrated by distinct signaling pathways that regulate their polarization and differentiation. Granulocyte macrophage-colony stimulating factor (GM-CSF) and macrophage-colony stimulating factor (M-CSF) are key determinants of monocyte fate, driving differentiation toward pro-inflammatory M1 and anti-inflammatory M2 phenotypes, respectively [110,131]. GM-CSF, IFN-γ, and LPS are principal inducers of M1 polarization, promoting the release of IL-12 and IL-23 as well as CXCL9/10, thereby amplifying inflammatory responses [132,133]. In contrast, M-CSF, IL-4, and IL-10 favor M2 differentiation, characterized by the secretion of CCL1, CCL17, CCL18, CCL22, and CXCL13 which mediate immune suppression, angiogenesis, and tissue remodeling [134,135]. Furthermore, signaling pathways such as STAT3, NF-κB, and peroxisome proliferator activated receptor-γ (PPARγ) are involved in regulating the phenotypic transition of MoDMs. For instance, NF-κB activation promotes M1 polarization and pro-inflammatory responses, whereas PPARγ activation induces M2 polarization, enhancing tissue repair functions [124,131,136]. The balance between these pathways is critical in determining whether MoDMs contribute to inflammation or facilitate tissue repair. Moreover, crosstalk between MoDMs and glial cells (e.g., microglia and astrocytes) within the ischemic microenvironment further enhances their reparative function. This reprogramming mechanism is essential for resolving inflammation and mitigating long-term neurological damage [128].

MoDMs are key players in both acute inflammation and tissue repair after CIRI. Their recruitment and polarization are regulated by pathways involving GM-CSF, CCL2, NF-κB, and PPARγ. Elucidating how these pathways regulate MoDM function and phenotype transitions offers valuable insights into therapeutic strategies to attenuate neuroinflammation and promote recovery after ischemic stroke.

### 5.3. T Cells

Following ischemic stroke, T cells—particularly CD4+ T cells—are rapidly recruited to the injury site, where they become activated and differentiate into distinct subsets. These subsets (primarily Th cells and Tregs) exert distinct functions in regulating inflammation and orchestrating tissue repair [43].

CD4+ Th cells are commonly classified into subtypes such as Th1, Th2, Th17, and other specialized phenotypes [137,138]. Driven by IL-12 signaling, Th1 cells produce IFN-γ and TNF-α, thereby amplifying neuroinflammation and exacerbating ischemic injury [43]. Th17 cells differentiate in response to IL-6 and IL-23, secrete IL-17 together with TGF-β, and contribute to inflammatory damage, BBB disruption, and neuronal death during stroke [139]. In contrast, Th2 cells and Tregs exert predominantly anti-inflammatory and reparative functions. Under IL-2 and IL-4 signaling conditions, Th2 cells release IL-4, IL-5, IL-9, and IL-13, which help to resolve inflammation and facilitate tissue repair [44,111]. Tregs, which are characterized by expression of the transcription factor forkhead box P3, play a pivotal role in immune homeostasis by secreting IL-10 and TGF-β, thereby limiting excessive immune activation and promoting recovery [43,62].

The recruitment and differentiation of T cells in the ischemic brain are tightly regulated by chemokines and cytokines released from injured tissue [140]. A key mediator in this process is CCL2, which binds to CCR2 receptors on monocytes and T cells to direct their migration to the ischemic site [140]. Upon entering the brain, T cells differentiate into distinct subsets in response to local cytokines, including GM-CSF, IL-4, and others [43,141]. These differentiation processes are regulated by key signaling pathways, including NF-κB, STAT3, and JAK/STAT, that orchestrate Th cell polarization and determine the adoption of either a pro-inflammatory (Th1/Th17) or anti-inflammatory (Th2/Treg) phenotype [111,139,142].

T cells orchestrate both inflammatory and reparative responses in CIRI. The opposing roles of Th1/Th17 cells and Tregs highlight the complexity of immune regulation. Enhancing Treg function while suppressing pro-inflammatory T cells offers a promising therapeutic approach. Future studies should clarify the molecular mechanisms governing T cell differentiation and migration to enable targeted interventions.

### 5.4. Platelets

Platelets act as key mediators of both thrombosis and neuroinflammation [143]. Although traditionally associated with hemostasis, platelets have increasingly been implicated in the pathophysiology of ischemic stroke because of their role in inflammation. Platelets are rapidly activated following ischemic injury, leading to aggregation and thrombus formation that obstruct microvascular flow and exacerbate tissue injury [109,144]. This process contributes to both the acute phase of injury and the subsequent neuroinflammatory response.

Platelets contribute to the progression of CIRI by releasing soluble mediators that modulate both the coagulation cascade and the inflammatory response. Key mediators include serotonin, ADP, and thromboxane A_2_ [145,146]. These platelet-derived factors not only promote thrombus formation, but also facilitate the recruitment of peripheral immune cells to the ischemic site [147]. This thrombo-inflammatory response amplifies the neuroinflammatory cascade, further disrupting the BBB and exacerbating neuronal injury [143]. Studies have demonstrated that platelet-mediated inflammatory amplification exacerbates ischemic injury and impairs normal brain function [109]. Additionally, platelets release cytokines (e.g., IL-1β) which further activate microglia and astrocytes, sustaining a pro-inflammatory cycle that markedly worsens neuronal injury [101].

Furthermore, platelets play a crucial role in interacting with peripheral immune cells—particularly T lymphocytes—during CIRI. Evidence indicates that platelets regulate T cell activation and differentiation, thereby modulating the immune response to ischemic injury. Specifically, Th1 and Th17 T cell subsets contribute to tissue damage by producing pro-inflammatory cytokines, including IFN-γ and IL-17 [148]. These effects are mediated by platelet-induced endothelial activation, which upregulates adhesion molecules and facilitates T cell extravasation into the brain parenchyma [143,146,148]. Recruited T cells further activate microglia and amplify the inflammatory response. Conversely, Tregs, which are essential for limiting inflammation, interact with platelets to suppress excessive immune activation, thereby promoting tissue repair and reducing injury [147].

Recent studies have highlighted the therapeutic potential of targeting platelet-mediated pathways in CIRI. Inhibition of platelet activation using glycoprotein IIb/IIIa inhibitors or targeting glycoprotein Ibα has been shown to reduce infarct volume and improve neurological outcomes in experimental stroke models [51]. These findings suggest that modulating platelet function may represent a promising strategy to attenuate ischemic injury and promote neurological recovery. Furthermore, inhibiting platelet–T cell interactions has been proposed as an effective strategy to limit T cell-mediated injury, thereby reducing ischemic damage and improving clinical outcomes [149].

## 6. The Interaction Between Cerebral Cellular and Peripheral Immune Cells in Driving Inflammatory Cascade Responses

As discussed in the preceding sections, the immune system plays a pivotal role in the pathophysiology of CIRI, with both cerebral cellular and peripheral immune cells significantly influencing tissue damage and recovery. Resident CNS cells, including microglia and astrocytes, are essential for maintaining brain homeostasis under physiological conditions. However, following ischemic injury, these cells become activated and initiate a robust inflammatory response. This activation leads to the release of pro-inflammatory cytokines such as TNF-α, IL-1β, and IL-6, which drive neuroinflammation, aggravate neuronal injury, and disrupt the BBB [150]. These cytokines not only exacerbate the initial injury but also recruit peripheral immune cells to the injury site, thereby amplifying the inflammatory cascade and worsening tissue damage. Microglia, the resident macrophages of the brain, undergo substantial phenotypic changes in response to ischemic injury. In response to detecting DAMPs released by injured neurons, microglia rapidly transit from the resting form to the pro-inflammatory M1 phenotype [150]. In this activated state, microglia release a variety of inflammatory mediators—including TNF-α, IL-1β, and ROS—which promote local tissue injury and attract peripheral immune cells to the injury site [151]. Wu et al. demonstrated that microglia activate pattern recognition receptors (PRRs) such as TLRs, leading to chemokine release and recruitment of circulating monocytes and neutrophils into the ischemic brain [151]. These peripheral immune cells amplify the inflammatory response and activate other resident brain cells, perpetuating a vicious cycle of neuroinflammation and neuronal injury [129]. In addition to microglial activation, astrocytes play a crucial role in the immune response to CIRI. Following ischemic injury, reactive astrocytes secrete cytokines, chemokines, and extracellular matrix components, which regulate immune cell recruitment and polarization [66,152]. Cao et al. demonstrated that reactive astrocytes secrete pro-inflammatory cytokines including IL-6, which enhances the activation of microglia and peripheral immune cells, thereby amplifying neuroinflammation and aggravating tissue injury [153]. Moreover, astrocytes modulate BBB integrity by altering the expression of adhesion molecules, thereby increasing vascular permeability and facilitating the infiltration of peripheral immune cells, including neutrophils, monocytes, and T lymphocytes [74].

**Figure 2:**
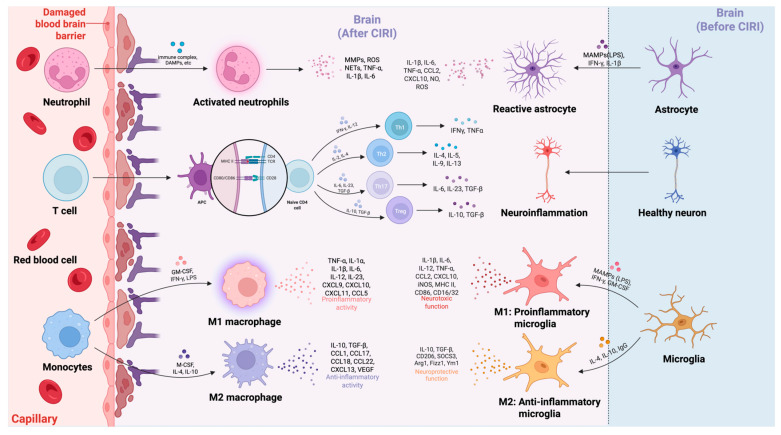
**Immune cell dynamics in CIRI.** The immune responses following BBB disruption in CIRI. Peripheral immune cells—including neutrophils, CD4^+^ T cells, and monocytes—infiltrate the ischemic brain, while resident microglia and astrocytes undergo activation. Microglia and MoMDs polarize into M1 and M2 phenotypes, contributing to either neuroinflammation or tissue repair (the figure is generated via BioRender).

The interaction between resident CNS cells and infiltrating peripheral immune cells is complex and bidirectional (Figure 2). While microglia and astrocytes initiate the inflammatory response, peripheral immune cells further exacerbate tissue damage. Neutrophils are the earliest peripheral immune cells to infiltrate the ischemic brain, guided by chemokines such as IL-8 and CXCL1. These cells secrete ROS and proteases, which directly damage neurons and disrupt the BBB, thereby worsening ischemic injury [66]. In addition, neutrophils release pro-inflammatory cytokines that activate other immune cells, perpetuating the inflammatory cascade. Monocytes infiltrating the brain differentiate into macrophages and polarize into either M1 or M2 phenotypes depending on the local microenvironment. M1 macrophages promote neuroinflammation by secreting cytokines such as IL-1β and ROS, whereas M2 macrophages facilitate tissue repair and inflammation resolution through the release of anti-inflammatory cytokines, including IL-10 and TGF-β [150]. The recruitment and activation of T lymphocytes—particularly CD4+ T cells—also play a pivotal role in the inflammatory response in CIRI. Th1 and Th17 subsets exacerbate ischemic injury by producing pro-inflammatory cytokines such as IFN-γ and IL-17, which activate microglia and macrophages, thereby amplifying inflammation and neuronal injury [66,137]. On the other hand, Tregs modulate the immune response by suppressing pro-inflammatory T cell activation and promoting tissue repair [138]. The recruitment of Tregs to the ischemic brain is essential for limiting excessive inflammation and preventing further neuronal injury [150,151]. Tregs play a dual role in CIRI by resolving inflammation and promoting tissue regeneration, thereby facilitating recovery.

Recent advances in single-cell RNA sequencing (scRNA-seq) have provided unprecedented resolution in characterizing the interactions between resident and peripheral immune cells in CIRI. These studies have identified stage-specific microglial subclusters with distinct inflammatory and reparative phenotypes [154] and uncovered key regulators such as Ifi27l2a [155] and Lrg1 [156] that modulate microglial and astrocytic responses following ischemic injury. Single-nucleus RNA sequencing has further delineated glial cell type-specific adaptations, revealing astrocyte heterogeneity and diverse microglial activation states [157]. In addition, scRNA-seq analyses of brain and blood compartments have captured the dynamic infiltration of neutrophils and T lymphocytes, linking peripheral immune entry to local neuroinflammatory cascades [31,158]. These findings highlight the capacity of scRNA-seq to resolve the cellular and molecular complexity of CIRI and to uncover novel targets for translational intervention.

Cerebral cellular and peripheral immune cell interactions are central to the immune response in CIRI. While pro-inflammatory cells exacerbate injury and BBB disruption, regulatory cells like Tregs and M2 macrophages promote repair. Recent scRNA-seq studies have further delineated microglial heterogeneity, astrocytic adaptations, and peripheral immune infiltration, identifying regulators such as Ifi27l2a and Lrg1. Collectively, these insights highlight the complexity of immune regulation in CIRI and its critical impacts on disease progression and recovery.

## 7. The Involvement of the Complement System and Inflammasomes in CIRI

The complement system and inflammasomes serve as critical mediators of neuroinflammation in CIRI. Activation of the complement cascade facilitates leukocyte infiltration, disrupts the BBB, and exacerbates neuronal injury [159]. Concurrently, inflammasomes such as NLRP3, AIM2, and NLRP6 drive the maturation of pro-inflammatory cytokines and induce pyroptosis [89,160]. Collectively, these pathways amplify sterile inflammation and are considered attractive targets for therapeutic intervention in CIRI.

### 7.1. The Complement System

The complement system is a major contributor to the pathophysiology of cerebral CIRI, playing a central role in neuroinflammation and neuronal damage. Complement cascade activation occurs rapidly following ischemic insult and reperfusion, with complement component C3 acting as a central mediator in all three pathways: classical, alternative, and lectin [159]. C3 cleavage produces the effector molecules C3a and C3b, which initiate downstream inflammatory responses. C3a, a potent pro-inflammatory anaphylatoxin, promotes the recruitment of immune cells such as neutrophils and monocytes to the ischemic region, thereby amplifying inflammation [159,161]. In contrast, C3b functions as an opsonin, enhancing microglial activation and promoting the phagocytosis of stressed neurons, ultimately contributing to neuronal apoptosis [161]. The terminal complement complex (C5b-9) exacerbates neurovascular injury by disrupting the BBB and inducing endothelial apoptosis [162]. Experimental studies have shown that complement inhibition at various stages effectively reduces infarct size and improves neurological outcomes in CIRI models. For instance, siRNA-loaded nanoparticles targeting C3 reduced its expression in microglia, suppressed inflammatory cascades, and decreased neuronal apoptosis [161]. Similarly, C3a receptor (C3aR) antagonists suppressed microglial activation, inhibited neutrophil infiltration, and preserved BBB integrity, underscoring their therapeutic potential [163].

In addition to its acute involvement in inflammation, the complement system also contributes to chronic neuroinflammation and delayed neuronal death [164]. Persistent activation of complement components sustains oxidative stress and cytokine production, creating a feedforward inflammatory loop that hinders tissue repair [159]. Moreover, crosstalk occurs between complement activation and other signaling pathways (e.g., NF-κB), further amplifying the production of pro-inflammatory cytokines such as IL-6 and TNF-α, and thereby exacerbating secondary brain injury [163,165].

Given the multifaceted role of the complement system in CIRI, targeting its activation represents a promising therapeutic strategy for attenuating neuroinflammation and preserving neurological function. Strategies such as the use of complement inhibitors, C3aR antagonists, and nanoparticle-based siRNA delivery systems have shown promising results in preclinical models, paving the way for future translational applications.

### 7.2. Inflammasomes

The roles of inflammasomes in CIRI have been extensively investigated, underscoring their central involvement in neuroinflammation and neuronal injury. Among the various inflammasomes, NLRP3 has received the most attention due to its pivotal role in the pathophysiology of CIRI. Structurally, the NLRP3 inflammasome is a multiprotein complex composed of the sensor protein NLRP3, the adaptor apoptosis-associated speck-like protein containing a CARD (ASC), and pro-caspase-1 [166]. Upon activation, these components assemble into a functional inflammasome that cleaves pro-caspase-1 into active caspase-1, which subsequently promotes the maturation and secretion of pro-inflammatory cytokines IL-1β and IL-18 [160,167].

**Figure 3:**
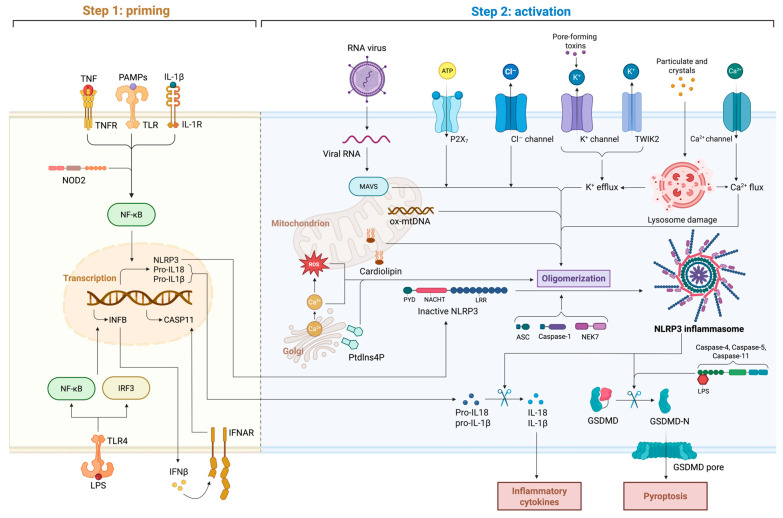
**NLRP3 signaling pathway and its implications in neuroinflammation.** The activation mechanism of the NLRP3 inflammasome in response to diverse pathological stimuli, leading to neuroinflammation and cell death. NLRP3 activation is initiated by multiple upstream signals, including mitochondrial dysfunction, ion flux dysregulation, and lysosomal destabilization, which collectively promote the assembly of the inflammasome complex with ASC and caspase-1. Subsequent activation of caspase-1 facilitates the maturation and secretion of pro-inflammatory cytokines IL-1β and IL-18, thereby amplifying the inflammatory cascade. In parallel, caspase-1-mediated cleavage of GSDMD triggers pyroptosis, further exacerbating neuronal injury (the figure is generated via BioRender).

NLRP3 inflammasome activation during CIRI is a tightly regulated, multistep process comprising priming and activation phases (Figure 3). The priming phase is initiated when PRRs, such as TLRs, recognize DAMPs or PAMPs [166]. This interaction activates the NF-κB signaling pathway, leading to transcriptional upregulation of NLRP3, pro-IL-1β, and pro-IL-18 [160,166]. In addition, nucleotide-binding oligomerization domain-containing protein-2 (NOD2), a cytosolic pattern recognition receptor, enhances NF-κB-mediated transcription after sensing muramyl dipeptide [168]. Meanwhile, the transcription factor interferon regulatory factor-3 (IRF3), which is activated downstream of TLR4 and cGAS–STING signaling, induces type I interferons that act through the IFNAR receptor, reinforcing the transcriptional priming of inflammasome components [160]. This preparatory step ensures the availability of inflammasome components that are necessary for subsequent activation. The activation phase is triggered by a variety of cellular stress signals prevalent during CIRI, including mitochondrial dysfunction, ROS production, potassium efflux, lysosomal rupture, and calcium flux. Among these factors, mitochondrial dysfunction plays a particularly critical role. Mitochondrial damage during CIRI results in the release of mitochondrial ROS, cardiolipin, and mtDNA into the cytosol [169]. These mitochondrial signals directly bind to NLRP3, inducing conformational changes that prime it for oligomerization [160,170,171]. Viral RNA can also activate NLRP3 indirectly through the mitochondrial antiviral signaling (MAVS) protein, linking antiviral responses with inflammasome activation [168]. Ionic fluxes represent another major class of activation signals. Extracellular ATP activates the purinergic receptor P2X7, driving K^+^ efflux [172]. Chloride efflux through Cl^−^ channels and K^+^ flux via the two-pore domain channel TWIK2 further contribute to ionic disequilibrium that facilitates inflammasome assembly [172]. Excessive calcium influx, often resulting from glutamate-induced excitotoxicity, provides an additional amplification signal [167]. Lysosomal rupture caused by accumulated cellular debris releases cathepsins into the cytosol, providing another potent trigger for inflammasome activation [170,171]. Importantly, the serine/threonine kinase NIMA-related ninase-7 (NEK7) directly binds to the NACHT domain of NLRP3 under conditions of K^+^ efflux, serving as an indispensable structural partner for inflammasome oligomerization [173]. These converging stimuli highlight the central role of NLRP3 as a key regulatory node in the inflammatory cascade of CIRI. Upon activation, NLRP3 oligomerizes and recruits ASC via pyrin domain interactions, forming cytosolic ASC specks. ASC then recruits pro-caspase-1 via CARD–CARD interactions, enabling its autoproteolytic cleavage into active caspase-1 [174]. Activated caspase-1 cleaves pro-IL-1β and pro-IL-18 into their mature, bioactive forms, which are secreted to amplify the inflammatory response [174,175]. Additionally, caspase-1 cleaves gasdermin D (GSDMD), generating an N-terminal fragment that forms membrane pores. This process induces pyroptosis—a highly inflammatory form of programmed cell death—thereby exacerbating neuroinflammation, BBB disruption, and neuronal injury during CIRI [169,176].

Although NLRP3 is the most extensively studied inflammasome in CIRI, other inflammasomes (including AIM2 and NLRP6) have also been implicated. The AIM2 inflammasome, which is activated by cytosolic double-stranded DNA, contributes to neuronal death and long-term cognitive impairment after ischemic stroke [89]. Similarly, the NLRP6 inflammasome, activated via interaction with the deubiquitinating enzyme BRCA1/BRCA2-containing complex subunit 3 (BRCC3), promotes neuroinflammation and pyroptosis, thereby exacerbating CIRI pathology [175].

Modulating inflammasome activation represents a promising therapeutic strategy for alleviating CIRI. Pharmacological inhibitors of the NLRP3 inflammasome, such as MCC950, have shown significant neuroprotective effects by reducing infarct volume, neurological deficits, and pro-inflammatory cytokine levels [170]. Additionally, targeting upstream regulators such as the TLR4/NF-κB signaling pathway and mitochondrial ROS production has demonstrated potential to inhibit inflammasome activation and its downstream consequences [170]. Beyond NLRP3, inflammasomes have also been shown to influence microglial polarization. Inflammasome signaling modulates the dynamic balance between M1 and M2 microglial phenotypes, which is essential for resolving inflammation and promoting neural repair [177].

In summary, inflammasomes—particularly NLRP3—play a central role in the inflammatory pathology of CIRI by mediating neuroinflammation, cytokine release, and pyroptosis. A deeper understanding of the mechanisms underlying inflammasome activation may facilitate the development of targeted therapies to mitigate the harmful effects of CIRI and improve clinical outcomes in ischemic stroke patients.

## 8. Therapeutic Strategies and Clinical Applications of Immune Regulation in CIRI

Therapeutic strategies for cerebral CIRI have increasingly focused on immune modulation, due to its important role in exacerbating neuronal injury and affecting recovery. The complexity of immune responses in CIRI indicates that targeting both pro- and anti-inflammatory pathways may represent a promising therapeutic strategy. Adjusting immune cell activity and related signaling pathways could mitigate neuroinflammation and potentially support functional recovery following ischemic injury.

One key therapeutic strategy in CIRI is to target inflammatory cytokines. For instance, Zhang et al. demonstrated that IL-11—a member of the cytokine IL-6 family—exerts neuroprotective effects in ischemic stroke by suppressing pro-inflammatory cytokines such as TNF-α and IL-1β, while enhancing the expression of anti-inflammatory cytokines like IL-10 [178]. This cytokine modulation reduces infarct volume and enhances neurological function after CIRI. Additionally, Wang et al. found that natural compounds such as kaempferol target oxidative and inflammatory stress pathways. By modulating key proteins including nuclear factor erythroid 2-related factor 2 and NF-κB, kaempferol reduces oxidative stress and apoptosis, further underscoring its therapeutic potential in CIRI [179].

Microglial polarization represents another promising therapeutic approach. Microglia, the resident immune cells of the brain, can adopt a pro-inflammatory M1 phenotype that exacerbates neuroinflammation or an alternatively activated M2 phenotype that promotes tissue repair and neuroprotection [180]. Strategies that promote the shift from the M1 to M2 phenotype have demonstrated therapeutic potential. For instance, Li et al. demonstrated that curcumin facilitates M1-to-M2 polarization, thereby reducing inflammation and improving motor function in CIRI models [181]. Similarly, Li et al. reported that loureirin B modulates microglial polarization through the STAT6/NF-κB signaling pathway, alleviating tissue damage and enhancing functional recovery [86]. Furthermore, Tat-NTS peptides target Annexin A1 in microglia and induce SUMOylation, thus promoting an anti-inflammatory phenotype, reducing neuronal apoptosis, and facilitating post-ischemic recovery [14].

Nanomedicine has emerged as a promising strategy to enhance the precision and efficacy of treatments for CIRI. While overcoming the BBB remains a significant challenge, advances in nanocarriers enable targeted drug delivery to ischemic brain regions [76]. For example, Sun et al. developed a smart liposomal nanocarrier that targets ischemic regions using ROS-responsive polymers and fibrin-binding peptides. This system, encapsulating Cl-amidine, reduces NET formation and inhibits the cGAS-STING signaling pathway, both of which contribute to neuroinflammation and neuronal injury [182]. Similarly, Wang et al. introduced a sequentially targeted nanomedicine using tannic acid and melanin-modified nanoparticles to protect neuronal mitochondria from oxidative stress and modulate immune responses, thereby reducing infarct size and enhancing post-ischemic recovery [183]. Additionally, extracellular vesicles derived from neural progenitor cells represent another novel immunomodulatory strategy. Engineered with targeting ligands such as RGD peptides, these extracellular vesicles efficiently accumulate in ischemic brain regions where they suppress microglial activation and pro-inflammatory cytokine release, thereby promoting recovery in ischemic stroke models [184].

In conclusion, immune modulation is expected to become a promising strategy for the treatment of CIRI. Strategies targeting inflammatory cytokines, microglial polarization, and oxidative stress pathways, combined with innovations in nanomedicine and extracellular vesicles, offer effective means to improve functional recovery.

## 9. Future Perspectives and Summary

Immune modulation in CIRI holds significant promise for the development of innovative, personalized, and adaptive therapeutic strategies. While some progress has been made in characterizing the functions of distinct immune cell populations, a comprehensive understanding of their coordinated interactions remains crucial for developing effective interventions. Particular attention should be directed toward the phenotypic plasticity of immune cells, with microglia and astrocytes being the most notable examples considering that they serve as central regulators of both neuroinflammatory and neuroprotective processes. These glial cells undergo dynamic phenotypic transitions in response to ischemic stress. Future studies should aim to elucidate the molecular signaling pathways and transcriptional networks that govern their activation and reprogramming, thereby informing therapeutic strategies that enhance their reparative and neuroprotective capacities while attenuating detrimental inflammatory responses [98,185].

The complex bidirectional crosstalk between resident and peripheral immune cells warrants further investigation. Infiltrating T lymphocytes, neutrophils, and monocytes contribute to parenchymal injury through direct interactions with neurons and glial elements. Deciphering the molecular cues that orchestrate their recruitment and effector functions may reveal novel therapeutic avenues to mitigate secondary damage [186]. Beyond the acute phase, emphasis should also be placed on the reparative phase of the immune response. Regulatory T cells and M2-polarized macrophages play essential roles in promoting immune resolution and tissue regeneration. Unraveling the mechanisms underlying immune tolerance and inflammation resolution may facilitate the development of dual-function therapeutic approaches that concurrently suppress pathological inflammation and foster neurorestorative processes [44].

Furthermore, extracellular vesicles—particularly exosomes—have emerged as compelling modulators of immune responses in CIRI. These nanoscale vesicles, which are secreted by neural and immune cells, transport a diverse repertoire of molecular cargo capable of modulating intercellular communication. Growing evidence suggests that exosomes can influence immune cell behaviors and confer neuroprotection, highlighting their potential as both biomarkers and therapeutic delivery systems [12,187,188]. In parallel, advancements in genomics and multi-omics technologies have enabled the characterization of individual immunologic profiles, thereby laying the foundation for precision immunotherapy. This approach allows for patient-specific modulation of immune responses, optimizing therapeutic efficacy while minimizing off-target effects [189,190].

Collectively, CIRI involves a multifaceted cascade of immune responses that profoundly influence the extent of neuronal injury and subsequent tissue repair. Both resident and peripheral immune cells contribute to this pathophysiological process, with microglia and astrocytes assuming pivotal, bidirectional roles in the initiation and resolution of inflammation. While excessive or prolonged immune activation exacerbates ischemic brain injury, timely and regulated immune responses can facilitate tissue regeneration, neurogenesis, and functional recovery. The intrinsic adaptability of the immune system underscores its therapeutic potential when appropriately modulated to shift from a pro-inflammatory to a reparative phenotype.

Emerging therapeutic strategies, such as the modulation of cytokine signaling networks, regulation of microglial polarization, inhibition of inflammasome activation, and nanomedicine-enabled targeted drug delivery, offer new opportunities for clinical intervention. Additionally, exosome-based therapies and precision medicine approaches tailored to individual immune signatures hold great potential for enhancing the specificity of treatments and improving clinical outcomes. Moving forward, research should continue to focus on elucidating the mechanisms that regulate immune cell activation, migration, and phenotypic plasticity, with particular emphasis on the signaling interactions between resident and infiltrating immune populations. Ultimately, advancing our understanding of CIRI immunopathology is expected to support the development of novel therapeutic paradigms aimed at attenuating acute ischemic injury and promoting long-term neurological recovery, thereby offering renewed hope for patients with ischemic stroke.

## Data Availability

Not applicable.
